# Association of Neighborhood Social Vulnerability With Metastatic Cancer at Diagnosis

**DOI:** 10.1002/cam4.71426

**Published:** 2026-01-18

**Authors:** Muhammad Sohaib Khan, Tammy Leonard, Sean Young, Natalie Williams, Jennie Meier, Gilbert Z. Murimwa, Herbert J. Zeh, Patricio M. Polanco

**Affiliations:** ^1^ Department of Surgery UT Southwestern Medical Center Dallas Texas USA; ^2^ Peter O'Donnell Jr. School of Public Health UT Southwestern Medical Center Dallas Texas USA

**Keywords:** delayed diagnosis, disparities, insurance status, metastatic cancer, social vulnerability

## Abstract

**Background:**

Relationships between socioeconomic factors and metastatic cancer at diagnosis have not been well studied. Using CDC's Social Vulnerability Index (SVI) we studied the association of metastatic cancer at initial diagnosis with 16 social factors and their interaction with insurance status.

**Methods:**

California and Texas cancer registries, merged with the SVI database, were used to identify adult patients diagnosed with breast, colorectal, liver, lung, ovarian, pancreatic, or prostate cancer from 2015 to 2019. To determine the association of SVI with metastatic cancer at initial diagnosis, multivariable binary logistic regression analyses were performed.

**Results:**

Of the 654,016 patients included, 149,476 (21.5%) were diagnosed with metastatic cancer at diagnosis. Overall, the adjusted odds of metastasis at diagnosis increased by 5% for every 10 unit increase in SVI. Stratified by cancer type, the odds (95% confidence interval) of metastatic cancer at diagnosis were: breast 1.04 (1.03–1.05), colorectal 1.01 (1.01–1.02), liver 1.03 (1.02–1.05), lung 1.01 (1.01–1.02), pancreatic 1.02 (1.01–1.03), prostate 1.06 (1.05–1.07). Interaction analysis of insurance with SVI revealed that the marginal effect of the association between SVI and the risk of metastasis at initial diagnosis increased most substantially as SVI increased for patients who had insurance. It was relatively constant for uninsured and Medicaid patients, who had the overall highest average risk.

**Conclusions:**

Increased neighborhood social vulnerability is associated with an increased risk of metastatic cancer at initial diagnosis. While uninsured patients or those on Medicaid had a higher risk, patients with other insurance types experienced the largest increases in risk associated with increasing SVI.

## Graphical abstract

Increased neighbourhood social vulnerability is associated with an increased risk of metastasis at diagnosis. While Medicaid and uninsured patients had higher risk, patients with other insurance experienced larger increases in risk associated with SVI.

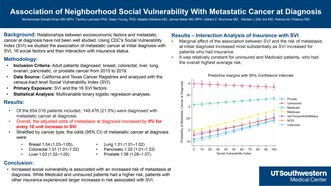

## 1 Introduction

Over 600,000 deaths result from cancer annually in the United States (US) making it the second most common cause of death [1]. Stage at diagnosis is one of the most important determinants of cancer survival [2]. While advancements in therapeutics have led to significant improvements for nonmetastatic cancers, survival after diagnosis of metastatic cancer remains dismal [3]. 5‐year survival rates range from 30% for stage IV breast and prostate cancers to less than 10% for metastatic pancreatic and lung cancers [3]. Early detection, therefore, offers the best chance for long‐term survival.

Early detection of cancer requires screening of asymptomatic patients and timely diagnosis of symptomatic patients. Screening is effective only for identifying preclinical breast, colon, lung, and cervical cancers. Timely diagnosis of patients who have symptoms requires patients to have self‐awareness and knowledge, access to a primary care physician and appropriate and timely diagnostic investigations [4, 5, 6]. Patients with limited access to health care, those lacking a primary care physician, and uninsured or under‐insured patients are less likely to be diagnosed with cancer at an early stage [6, 7, 8]. Additionally, these disparities have persisted even after the expansion of insurance coverage [9, 10]. It has been proposed that factors outside the health care system (e.g., other social determinants) may present additional barriers to timely diagnosis and treatment [11, 12]. Moreover, the interaction of these social determinants with insurance status has also not been fully explored.

An important measure of adverse social determinants of health experienced by populations is the Social Vulnerability Index (SVI), a tool developed by the Centers for Disease Control (CDC) [13]. Based on 16 social factors, SVI measures vulnerability at either the county or census tract level. It offers the opportunity to study the association of multiple social factors with cancer care and outcomes [11]. However, most studies performed in this area are inherently limited as the analyses have been performed at the county level [14]. Social determinants of health can vary widely within a county as evidenced by the fact that neighborhoods within the same county can have a difference in life expectancy of almost 26 years [15]. In contrast, census tracts are small geographical areas comprising an average population of 4000 inhabitants. SVI measured at the census tract level is more representative of neighborhood vulnerability factors. Moreover, no studies to date have examined the combined influence of SVI and health care access (measured by insurance status) on the risk of metastasis at diagnosis.

In this study we examine three aspects of the association between metastatic cancer at diagnosis and SVI to shed light on these relationships. First, the spatial patterning of social determinants of health within residential neighborhoods may create clusters of both elevated SVI and metastatic cancer at initial diagnosis. We use geospatial methods to examine whether clusters of metastatic cancer and SVI overlap. Next, we use SVI to measure cancer patients' exposure to social determinants of health and examine associations with metastatic cancer at diagnosis. Last, we examine whether associations between SVI and metastatic cancer vary by patient insurance status.

## 2 Methods

### 2.1 Setting and Data Source

This was a cross‐sectional study of adult patients diagnosed with breast, colorectal, liver, lung, ovarian, pancreatic, or prostate cancer from 2015 to 2019 in the US states of California and Texas. It was conducted following the approval of the University of Texas Southwestern Medical Center Institutional Review Board. The data were sourced from the California and Texas Cancer registries in combination with the SVI 2020 Data, which is based upon the American Community Survey data collected during 2016–2020. Data were merged using 2020 census tract Federal Information Processing Standards (FIPS) codes. These FIPS codes were derived using patients' residential longitudes and latitudes allowing us to merge patient data with the 2020 SVI data optimally covering the study period.

### 2.2 California and Texas Cancer Registries

Mandated by state law, these are population‐based statewide registries that attempt to include data for all patients diagnosed with cancer in California and Texas. Both meet the National Program of Cancer Registries high data standards, report to the NCI Surveillance Epidemiology and End Results (SEER) Program and are Gold Certified by the North American Association of Central Cancer Registries.

### 2.3 Social Vulnerability Index

SVI was developed by The Agency for Toxic Substances and Disease Registry's Geospatial Research, Analysis, and Services Program for CDC. The index was first published in 2011 [13], and is revised biennially, using the US Census Bureau's American Community Survey 5‐year estimates data. The SVI represents the geographic area's ranking compared to all other areas in the US with regard to overall social vulnerability based on 16 variables (Table S1) grouped into four themes: socioeconomic status household characteristics, racial and ethnic minority status (all racial and ethnic minorities are grouped), housing, and transportation. Data for each variable, theme, and SVI is available for each US State at either the census tract or county level as percentile rankings

### 2.4 Study Population and Patient Selection

Adult patients aged over 18 years and diagnosed with either breast, colorectal, liver, lung, ovarian, pancreatic, or prostate cancer were identified using the ICD‐O‐3 codes (Figure S1). We selected these cancers as they are the top five leading causes of cancer‐related deaths for male and female patients [16]. Patients who were reported to have cancer from death certificates and autopsy reports, those with in situ tumors, and missing stage or SVI information were excluded.

### 2.5 Dependent/Primary Outcome Variable

We used the SEER Summary staging category of ‘Distant,’ to define the presence of metastatic cancer at presentation. This staging system takes into consideration all information available within the first 4 months of diagnosis including the operative findings if applicable.

### 2.6 Independent Variables

The primary exposure is SVI. The SVI and the 16 SVI factors percentile rankings were used as a continuous variable. SVI was scaled so that it ranged from 0 to 10; the scaled SVI measure divided by 10 corresponds to a census tract's percentile ranking (i.e., SVI = 2 indicates a tract at the 20th percentile) and categorized using tertiles as either least, less, or most vulnerable neighborhoods. We also created a variable measuring the accumulated SVI risk across factors. “Accumulated SVI” is the number of factors for which an area ranked in the most vulnerable tertile. This measure ranged from 0 (no factors were in the most vulnerable tertile) to 16 (all 16 factors were in the most vulnerable tertile).

Additional covariates included age (categorized as 18–49 years, 50–64 years, 65–79 years or ≥ 80 years); gender (male, female, and non‐binary); US State (California or Texas); Non‐Hispanic White, Non‐Hispanic Black, Non‐Hispanic Asian, Non‐Hispanic and of other races, Hispanic of any race or unknown race/ethnicity; Insurance status (privately insured, uninsured, Medicaid, Medicare, insurance not specified (NOS), unknown or missing and combined category of Veterans Affairs (VA), Tricare, Indian Health Services (IHS), and Military). To control for population density, we used 2013 Rural–Urban Continuum Codes (RUCC) categorized as residential county in metropolitan areas of 1 million population or more (RUCC 1), in metropolitan areas with less than 1 million population (RUCC 2, 3) or in nonmetropolitan areas (RUCC 4–9) [17]. To control for healthcare utilization, we used the CDC's Population Level Analysis and Community Estimates (PLACES) 2020 dataset [18] for county‐level prevalence estimates for routine checkup within the past year among adults. These estimates are based on Behavioral Risk Factor Surveillance System 2017–2018 and American Community Survey 2014–2018 data.

### 2.7 Analysis

Descriptive statistics were performed followed by bivariate analysis using the chi‐square test for categorical variables and independent sample *T*‐test for continuous variables. To evaluate the spatial distribution of the relationship between SVI and cancer metastases, we performed univariate cluster detection using the local Moran's *I* test of spatial autocorrelation at the census tract level for both California and Texas. SVI and cancer metastatic rates were tested independently. Moran's *I* identifies both high and low clusters based on a statistical comparison of the hypothetical cluster with the expected distribution found in the larger dataset [19]. Clusters with pseudo‐*p* values < 0.1 and < 0.05 were retained as statistically significant. We then identified census tracts with overlapping clusters for both high SVI and high metastatic cancer rates, low SVI and low metastatic cancer rates, and mixed clusters (high SVI and low metastatic cancer rate, and vice versa) and mapped the resulting bivariate clusters [20]. Geospatial analysis and mapping were performed using ArcGIS Pro v3.3.1 (ESRI, Redlands, CA).

We used multivariable logistic regression to determine the relationship between SVI and the primary outcome. All models were adjusted for the covariates mentioned above but differed in how SVI was used for analysis. First, we combined all cancer types and used SVI as a continuous variable. In the same model we included the interaction between insurance status and SVI. Next, we stratified the model by cancer type. We then used the 16 SVI factors as continuous variables in an overall model and then stratified by cancer type. Finally, to determine if there is a cumulative effect whereby more SVI factors indicating a high degree of vulnerability indicate greater metastatic cancer risk, we examined associations with “accumulated SVI” described in the variable section. For statistical analysis StataCorp. 2023. Stata Statistical Software: Release 18 (College Station, TX: StataCorp LLC) was used with a *p* value < 0.05 considered significant.

## 3 Results

### 3.1 Descriptive and Bivariate Analysis

This study included 654,016 patients of which 378,897 (57.9%) were from California and 275,119 (42.1%) from Texas (Table 1). Breast cancer was the most common type of cancer (31.4%) while ovarian cancer was the least common (2.6%). Over 50% of patients with lung (51.8%), ovarian (54.1%), and pancreatic (52.8%) cancers were diagnosed with metastatic cancer at diagnosis. Patients diagnosed with metastatic cancer at diagnosis were more likely to be aged 80 years or more (32.4%), male (25.6%), from Texas (23.2%), Non‐Hispanic Black (25.2%), and uninsured (35.5%). Patients diagnosed with metastatic disease also had a significantly higher mean SVI score (49.9 ± 27.8 *p* < 0.001) (Table 1). Except for ovarian cancer, for all other cancer types, a significantly greater proportion of patients in the most vulnerable tertile group were diagnosed with metastatic cancer (Table S2).

**TABLE 1:** Patient characteristics with number and proportion of patients with metastasis.

Variables		Overall	Patients with metastasis at initial diagnosis	Patients without metastasis at initial diagnosis	*p*
		*n* = 654,016	*n* = 149,476	*n* = 504,540	
		(% of total)	(row %)	(row %)	
Age	Less than 50	68,926 (10.5)	11,350 (16.5)	57,576 (83.5)	< 0.001
	50–64	222,013 (33.9)	45,695 (20.6)	176,318 (79.4)	
	65–79	276,587 (42.3)	64,396 (23.3)	212,191 (76.7)	
	80 or more	86,490 (13.2)	28,035 (32.4)	58,455 (67.6)	
Gender	Male	295,730 (45.2)	75,837 (25.6)	219,893 (74.4)	< 0.001
	Female	358,164 (54.8)	73,614 (20.5)	284,550 (79.4)	
	Non‐binary	122 (0.02)	25 (20.5)	97 (79.5)	
State of diagnosis	California	378,897 (57.9)	85,740 (22.6)	293,157 (77.4)	< 0.001
	Texas	275,119 (42.1)	63,736 (23.2)	211,383 (76.8)	
Race/Ethnicity	Non‐Hispanic White	401,018 (61.3)	91,480 (22.8)	309,538 (77.2)	< 0.001
	Non‐Hispanic Black	6333 (9.7)	15,967 (25.2)	47,364 (74.8)	
	Non‐Hispanic Asian	58,161 (8.9)	13,688 (23.5)	44,473 (76.5)	
	Non‐Hispanic/Other races	6230 (0.95)	1393 (22.4)	4837 (77.6)	
	Hispanic	120,546 (18.4)	26,717 (22.2)	93,829 (77.8)	
	Unknown race/Ethnicity	4730 (0.7)	231 (4.9)	4499 (95.1)	
Insurance status	Private	246,110 (37.6)	43,625 (17.7)	202,485 (82.3)	< 0.001
	Uninsured	19,578 (2.9)	6947 (35.5)	12,631 (64.5)	
	Medicaid	48,850 (7.5)	14,239 (29.1)	34,611 (70.8)	
	Medicare	284,437 (43.5)	73,898 (25.9)	210,539 (74.0)	
	VA/Tricare/Military	10,172 (1.6)	2227 (21.9)	7945 (78.1)	
	Insurance NOS	22,804 (3.5)	4598 (20.2)	18,206 (79.8)	
	Unknown/Missing	22,065 (3.4)	3942 (17.9)	18,123 (82.1)	
Primary site	Breast	205,322 (31.4)	12,016 (5.8)	193,306 (94.1)	< 0.001
	Colorectal	108,793 (16.6)	25,415 (23.4)	83,378 (76.6)	
	Liver	25,893 (3.9)	4399 (17.0)	21,440 (82.9)	
	Lung	127,110 (19.4)	65,831 (51.8)	61,279 (48.2)	
	Ovarian	17,152 (2.6)	9269 (54.1)	7873 (45.9)	
	Pancreas	36,313 (5.5)	19,176 (52.8)	17,137 (47.2)	
	Prostate	133,497 (20.4)	13,370 (10.0)	120,127 (89.9)	
Rural Urban Continuum Codes (RUCC)	RUCC 1	358,236 (70.5)	102,753 (22.3)	358,236 (77.7)	< 0.001
	RUCC 2, 3	109,710 (21.9)	33,836 (23.6)	109,710 (76.4)	
	RUCC 6–9	36,594 (7.8)	12,887 (26.0)	36,594 (73.9)	
Mean prevalence of routine checkup within the past year in residential county ± standard deviation		71.9 ± 1.6	71.9 ± 1.6	71.9 ± 1.6	0.63
Mean SVI score ± standard deviation		46.6 ± 28.0	49.9 ± 27.8	45.7 ± 28.0	< 0.001

### 3.2 Geospatial Bivariate Analysis

**FIGURE 1:**
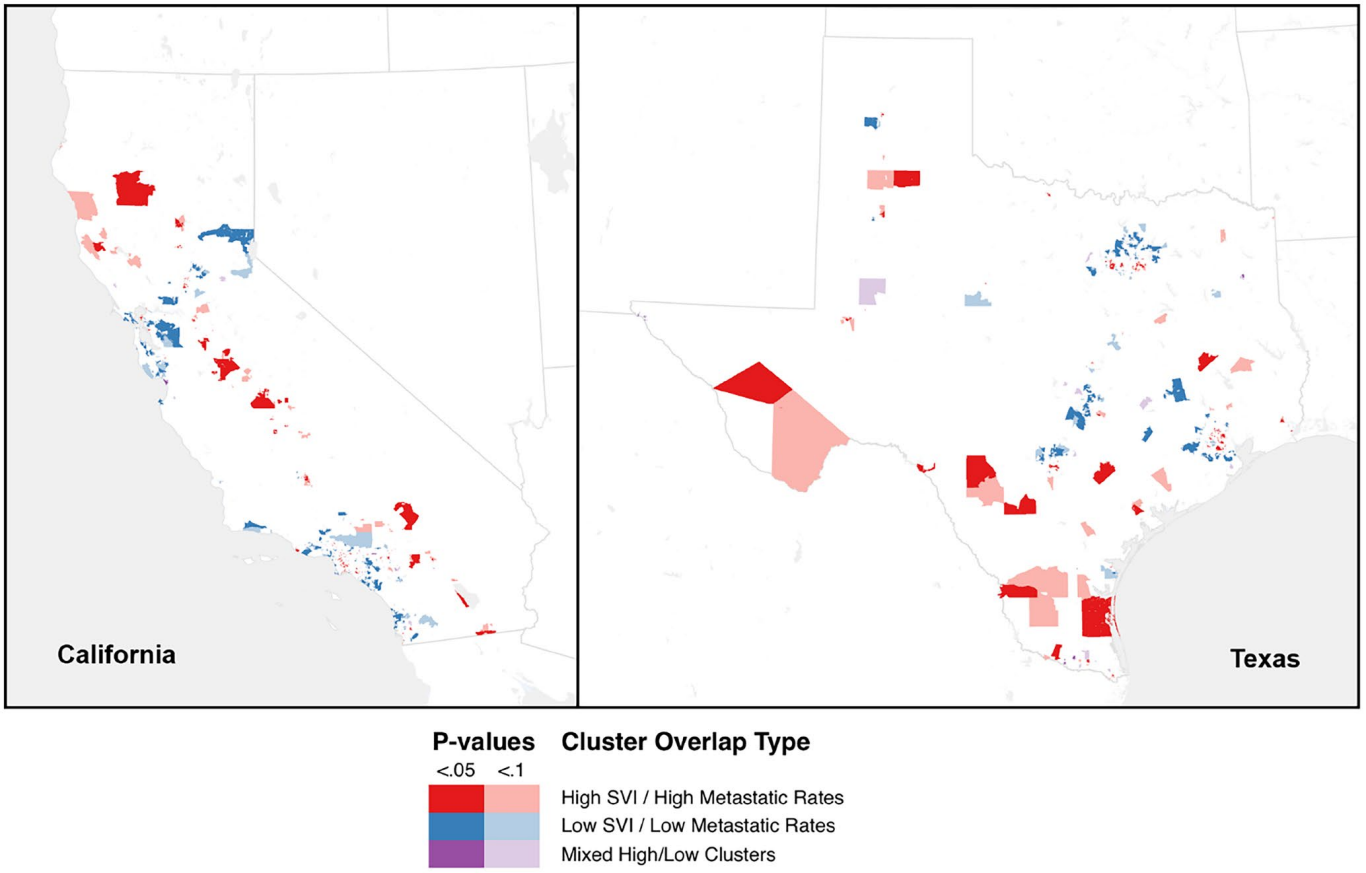
Map of overlapping clusters of proportions of patients with metastasis at diagnosis and SVI in California and Texas.

Overlapping geospatial clusters of high SVI and high metastatic rates (high clusters) as well as low SVI and low metastatic rates (low clusters) were identified in both California and Texas (Figure 1). In California, about 16.4% of census tracts (1479/9027 tracts) contained overlapping clusters, of which 50% (740 tracts) were low clusters, 44% (647 tracts) were high clusters, and 6% (92 tracts) were mixed high/low clusters. In Texas, about 17.1% of census tracts (1092/6389 tracts) contained overlapping clusters, of which 58% (634 tracts) were low clusters, 36% (393 tracts) were high clusters, and 6% (65 tracts) were mixed high/low clusters.

### 3.3 Multivariable Analysis

#### 3.3.1 SVI and Metastatic Cancer at Initial Diagnosis

**FIGURE 2:**
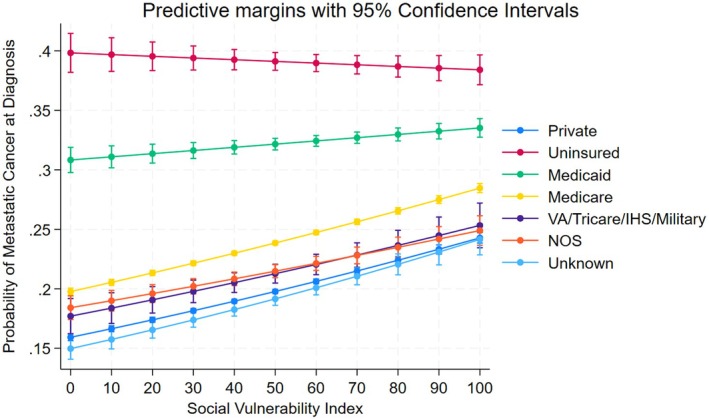
Plot of predictive margins with 95% confidence intervals of insurance status and SVI.

In the first multivariable model, we found that with every 10 percentile point increase in SVI, there was a 5% increase in the risk of metastatic cancer at diagnosis (Table 2). This association remained when the analysis was stratified by residential US State at the time of diagnosis (Tables S3 and S4) and after adjustment for the interaction between SVI and insurance status (Table 2). We examined the marginal effect of SVI on metastatic cancer risk for patients with different insurance types. While the odds were highest for uninsured or those on Medicaid, the odds increased more with each unit increase in SVI for patients with other insurance types (Figure 2). The probability of metastatic cancer was around 40% for patients lacking insurance across all levels of neighborhood SVI. For patients with Medicaid, the probability of metastatic cancer ranged from just over 30% (lowest SVI neighborhoods) to just under 35% (highest SVI neighborhoods). Patients with insurance types had a larger range of predicted probabilities. For example, patients with private insurance had on average predicted probabilities of metastatic cancer of 15% in the neighborhoods with lowest SVI, but this increased to nearly 25% in neighborhoods with the highest SVI. From the models stratified by cancer type (Table 3), except for ovarian cancer, the odds of primary outcome increased for all other cancer types as SVI increased. For each 10 unit increase in SVI the odds increased by 1%–6%. The 16 SVI factors model (Table S1) revealed that while multiple factors were associated with the primary outcome, none were prominently larger or consistent across cancer types. However, the aggregate variable model (Table 4) revealed that the odds of metastasis at diagnosis increased incrementally as the SVI factor vulnerabilities increased from 3 to 15.

**TABLE 2:** Multivariable logistic regression analysis for metastatic cancer at the time of diagnosis of all cancers combined.

Variables		Overall	Overall with interaction of SVI and insurance
SVI		1.05 (1.04–1.05)	1.05 (1.05–1.06)
Age	Less than 50	Ref	Ref
	50–64	1.23 (1.20–1.26)	1.22 (1.19–1.25)
	65–79	1.39 (1.36–1.43)	1.39 (1.35–1.42)
	80 or more	2.24 (2.18–2.30)	2.23 (2.17–2.29)
Gender	Male	Ref	Ref
	Female	0.76 (0.75–0.77)	0.76 (0.75–0.77)
	Non‐binary	0.72 (0.46–1.12)	0.71 (0.46–1.11)
State of diagnosis	California	Ref	Ref
	Texas	0.99 (0.98–1.01)	0.99 (0.98–1.01)
Race/Ethnicity	Non‐Hispanic White	Ref	Ref
	Non‐Hispanic Black	1.05 (1.03–1.08)	1.05 (1.03–1.07)
	Non‐Hispanic Asian	1.06 (1.04–1.09)	1.06 (1.04–1.08)
	Non‐Hispanic/Other races	0.99 (0.93–1.04)	0.98 (0.92–1.01)
	Hispanic	0.84 (0.27–0.85)	0.84 (0.83–0.86)
	Unknown race/Ethnicity	0.18 (0.16–0.21)	0.18 (0.16–0.21)
Insurance status	Private	Ref	Ref
	Uninsured	2.54 (2.46–2.62)	3.57 (3.32–3.83)
	Medicaid	1.87 (1.83–1.92)	2.38 (2.26–2.52)
	Medicare	1.28 (1.26–1.29)	1.30 (1.27–1.34)
	VA/Tricare/Military	1.10 (1.05–1.16)	1.14 (1.02–1.26)
	Insurance NOS	1.11 (1.08–1.15)	1.19 (1.11–1.28)
	Unknown/Missing	0.95 (0.92–0.99)	0.93 (0.86–1.00)
Insurance status interacted with SVI	Private	—	Ref
	Uninsured		0.94 (0.93–0.95)
	Medicaid		0.96 (0.95–0.97)
	Medicare		0.99 (0.99–1.00)
	VA/Tricare/Military		0.99 (0.97–1.01)
	Insurance NOS		0.98 (0.97–0.99)
	Unknown/Missing		1.00 (0.99–1.02)
Rural Urban Continuum Codes (RUCC)	RUCC 1	Ref	Ref
	RUCC 2, 3	1.01 (0.99–1.03)	1.01 (0.99–1.02)
	RUCC 6–9	1.13 (1.10–1.16)	1.12 (1.09–1.15)
Mean prevalence of routine checkup within the past year in residential county		0.99 (0.98–0.99)	0.99 (0.98–0.99)

**TABLE 3:** Multivariable logistic regression analysis for metastatic cancer at time of diagnosis of all cancers stratified by cancer type.

Variables		Breast	Colorectal	Liver	Lung	Ovary	Pancreas	Prostate
SVI		1.04 (1.03–1.04)	1.01 (1.01–1.02)	1.03 (1.02–1.05)	1.01 (1.01–1.02)	1.01 (0.99–1.02)	1.02 (1.01–1.03)	1.06 (1.05–1.07)
Age	Less than 50	Ref	Ref	Ref	Ref	Ref	Ref	Ref
	50–64	1.007 (0.96–1.06)	0.85 (0.81–0.89)	0.78 (0.67–0.90)	0.94 (0.87–1.01)	2.03 (1.87–2.21)	1.46 (1.33–1.60)	0.77 (0.67–0.89)
	65–79	0.87 (0.81–0.93)	0.73 (0.69–0.77)	0.85 (0.72–0.99)	0.74 (0.69–0.79)	3.53 (3.16–3.94)	1.42 (1.29–1.56)	1.09 (0.94–1.26)
	80 or more	1.38 (1.27–1.49)	0.75 (0.71–0.79)	0.96 (0.80–1.15)	0.82 (0.76–0.89)	4.84 (4.19–5.58)	1.32 (1.19–1.47)	5.41 (4.65–6.29)
Gender	Male	Ref	Ref	Ref	Ref	—	Ref	—
	Female	0.73 (0.61–0.87)	0.95 (0.92–0.97)	0.83 (0.77–0.90)	0.83 (0.82–0.85)		0.88 (0.85–0.92)	
	Non‐binary	1.39 (0.48–4.02)	0.20 (0.05–0.85)	0.35 (0.04–2.68)	0.59 (0.26–1.36)		1.54 (0.28–8.46)	
State of diagnosis	California	Ref	Ref	Ref	Ref	Ref	Ref	Ref
	Texas	1.04 (0.99–1.10)	1.05 (1.01–1.09)	1.07 (0.97–1.17)	0.92 (0.89–0.95)	0.87 (0.79–0.95)	0.96 (0.91–1.02)	0.94 (0.89–0.99)
Race and ethnicity	Non‐Hispanic White	Ref	Ref	Ref	Ref	Ref	Ref	Ref
	Non‐Hispanic Black	1.45 (1.36–1.54)	1.19 (1.13–1.25)	1.16 (1.04–1.31)	1.15 (1.10–1.19)	1.22 (1.07–1.39)	1.04 (0.96–1.12)	1.23 (1.16–1.30)
	Non‐Hispanic Asian	0.92 (0.86–0.99)	0.89 (0.85–0.94)	0.91 (0.81–1.02)	1.34 (1.28–1.39)	0.86 (0.77–0.96)	0.97 (0.90–1.05)	0.99 (0.92–1.08)
	Non‐Hispanic/Other	1.08 (0.90–1.29)	1.03 (0.89–1.19)	1.04 (0.78–1.39)	1.14 (1.01–1.29)	1.06 (0.75–1.47)	1.02 (0.82–1.27)	0.97 (0.79–1.19)
	Hispanic	0.88 (0.84–0.93)	0.94 (0.91–0.98)	0.89 (0.82–0.97)	1.25 (1.20–1.30)	0.95 (0.87–1.03)	0.99 (0.93–1.05)	0.24 (0.18–0.32)
	Unknown	0.65 (0.47–0.90)	0.27 (0.16–0.37)	0.83 (0.35–1.99)	0.58 (0.42–0.79)	0.45 (0.21–0.99)	0.58 (0.30–1.12)	1.05 (1.00–1.11)
Insurance status	Private	Ref	Ref	Ref	Ref	Ref	Ref	Ref
	Uninsured	3.34 (3.06–3.65)	1.77 (1.66–1.89)	1.53 (1.33–1.76)	1.70 (1.58–1.83)	1.19 (1.03–1.39)	1.44 (1.27–1.63)	4.22 (3.80–4.69)
	Medicaid	2.28 (2.14–2.42)	1.57 (1.49–1.65)	1.24 (1.12–1.38)	1.29 (1.22–1.35)	1.35 (1.20–1.49)	1.39 (1.27–1.52)	2.84 (2.61–3.08)
	Medicare	1.37 (1.29–1.46)	1.17 (1.12–1.22)	0.93 (0.85–1.02)	0.97 (0.95–1.004)	1.15 (1.05–1.27)	1.03 (0.97–1.09)	1.31 (1.24–1.38)
	VA/Tricare/Military	1.24 (1.02–1.51)	1.14 (0.99–1.29)	1.11 (0.87–1.41)	0.75 (0.69–0.81)	0.91 (0.64–1.31)	0.96 (0.79–1.16)	1.01 (0.88–1.16)
	Insurance NOS	1.14 (1.02–1.26)	1.07 (0.99–1.16)	0.91 (0.75–1.10)	1.001 (0.93–1.07)	1.17 (0.99–1.39)	1.09 (0.96–1.24)	1.04 (0.92–1.17)
	Unknown/Missing	1.53 (1.37–1.71)	1.28 (1.16–1.40)	1.29 (1.05–1.60)	1.23 (1.14–1.33)	1.29 (1.04–1.59)	1.33 (1.16–1.52)	0.52 (0.47–0.58)
Rural Urban Continuum Codes (RUCC)	RUCC 1	Ref	Ref	Ref	Ref	Ref	Ref	Ref
	RUCC 2, 3	1.05 (1.000–1.10)	0.97 (0.94–1.01)	1.17 (1.08–1.27)	1.01 (0.99–1.04)	1.002 (0.92–1.09)	0.99 (0.95–1.05)	0.89 (0.86–0.94)
	RUCC 6–9	1.28 (1.18–1.38)	1.01 (0.96–1.07)	1.36 (1.19–1.53)	1.07 (1.03–1.12)	1.003 (0.87–1.15)	1.11 (1.02–1.21)	1.05 (0.97–1.13)
Mean prevalence of routine checkup within the past year in residential county		0.97 (0.96–0.99)	0.98 (0.97–0.99)	0.99 (0.97–1.02)	0.98 (0.97–0.99)	1.01 (0.98–1.04)	0.99 (0.98–1.02)	0.97 (0.95–0.98)

**TABLE 4:** Association of the number of SVI factors in the most vulnerable tertile group with metastatic cancer: descriptive statistics with multivariable^a^ logistic regression analysis.

Number of SVI factors within most vulnerable tertile	Number of patients	Number of patients with metastatic cancer (%)	Odds^a^ ratios with confidence intervals
Patients with 0 SVI factors	17,609	3419 (19.4)	Ref
Patients with 1 SVI factors	73,113	14,025 (19.2)	0.96 (0.92–1.007)
Patients with 2 SVI factors	94,495	19,073 (20.2)	1.003 (0.96–1.04)
Patients with 3 SVI factors	96,047	20,889 (21.7)	1.08 (1.03–1.12)
Patients with 4 SVI factors	79,100	18,041 (22.8)	1.12 (1.07–1.17)
Patients with 5 SVI factors	59,634	14,145 (23.7)	1.17 (1.12–1.22)
Patients with 6 SVI factors	46,270	11,318 (24.5)	1.22 (1.17–1.27)
Patients with 7 SVI factors	40,676	10,332 (25.4)	1.27 (1.21–1.32)
Patients with 8 SVI factors	35,553	9099 (25.6)	1.29 (1.23–1.35)
Patients with 9 SVI factors	31,122	7849 (25.2)	1.26 (1.20–1.32)
Patients with 10 SVI factors	30,061	7819 (26.0)	1.31 (1.25–1.38)
Patients with 11 SVI factors	24,123	6486 (26.9)	1.37 (1.30–1.43)
Patients with 12 SVI factors	15,611	4185 (26.8)	1.35 (1.28–1.42)
Patients with 13 SVI factors	8454	2209 (26.1)	1.30 (1.22–1.38)
Patients with 14 SVI factors	1823	498 (27.3)	1.37 (1.23–1.53)
Patients with 15 SVI factors	306	87 (28.4)	1.47 (1.14–1.90)
Patients with 16 SVI factors	19	2 (10.5)	0.40 (0.09–1.79)

^a^ Model adjusted for age, race, gender, Hispanic ethnicity, insurance status, US State of Diagnosis, RUCC, mean prevalence of routine checkup within the past year in residential county.

#### 3.3.2 Demographic Factors

The odds of metastatic cancer at diagnosis increased with age (Tables 2 and 3). Compared to patients < 50 years, the odds for patients ≥ 80 years were highest for prostate cancer and for ovarian cancer: OR 4.84 (95% CI 4.19–5.58) (Table 3). Female patients had decreased odds across most cancer types. Compared with White patients, Black patients had increased odds across most cancer types; being 45% higher for breast cancer and 23% higher for prostate cancer. Compared with privately insured patients, uninsured patients and those on Medicaid had increased odds across all cancer types (Table 3). Compared to residence in metropolitan areas with a population of 1 million or more, residence in nonmetropolitan areas was associated with increased odds for most cancer types. The odds of metastatic cancer also decreased with each percent increase in the prevalence of routine checkup within the past year for most cancer types (Table 2, Tables S5 and S6).

## 4 Discussion

In this study we found that social vulnerability is associated with metastatic cancer at diagnosis for both screenable and non‐screenable cancers. By using novel geospatial techniques, we identified clusters of census tracts that had high SVI and high metastatic cancer rates and vice versa. Additionally, the odds of metastasis at diagnosis increased as more aspects of the census tract were ranked as being in the “most vulnerable” tertile. The marginal effect of higher SVI on the risk of metastasis at diagnosis increased more as SVI increased for insured patients compared to uninsured patients or those using Medicaid. This suggests that the impact of health insurance in reducing the risk of metastasis at diagnosis ameliorates in high SVI neighborhoods. Social vulnerability factors may deter symptomatic patients from seeking or receiving timely care until the cancer has metastasized even when they have health insurance.

The findings from this study align with prior studies which found significant associations of SVI with cancer screening rates, as well as mortality for screenable cancers [21, 22]. However, in this study the findings are applicable to both screenable and non‐screenable cancers. This indicates that screening alone cannot solve the problem of delayed diagnosis. The World Health Organization (WHO) distinguishes early diagnosis from screening defining it as “early identification of cancer in patients who have symptoms of the disease” [23]. This distinction is important as the two are different in their intended population, required resources and potential benefits and harms [23]. Early diagnosis is included in the National cancer plans of various countries like the National Health Service of England who in their “Long‐Term plan” included the goal of diagnosing 75% of cancer patients at an early stage [24]. The US National Cancer Plan has “Detect cancers early” as one of its goals; however, it appears to primarily focus on screening asymptomatic patients. Similarly, early diagnosis is missing from the goal of eliminating inequities in cancer care.

Timely diagnosis of cancer requires access to primary care physicians who play a critical role in referring patients for screening and investigating symptoms. A recent study reported that an annual primary care physician's visit was associated with a 39% reduction in the odds of metastatic cancer at diagnosis [6]. In this study we find that even after controlling for healthcare utilization, SVI was significantly associated with metastasis at diagnosis. In addition, insurance is considered to be important for access to healthcare [8, 25]. This has led to several interventions to expand insurance coverage such as, the National Breast and Cervical Cancer Early Detection Program and Medicaid expansion through the Affordable Care Act. Our study demonstrates that patients with Medicaid or no insurance have a higher risk of metastatic cancer compared to those with other insurance types. However, this risk gap narrows as patients with insurance experience increased risk in higher SVI neighborhoods. This suggests that the expansion of insurance coverage alone will not address disparities in diagnosis among patients living in vulnerable communities.

This study can potentially have significant positive implications. Firstly, by highlighting the disparities in diagnosis for seven different cancers, it supports the case for further studies and interventions that can improve early diagnosis of symptomatic patients. Several interventions have been found to be effective in improving rates of early diagnosis for symptomatic patients. These include pathways for rapid referral and standardized care, enhanced and coordinated diagnostic services and supporting primary and multidisciplinary care [26, 27, 28, 29, 30, 31, 32]. For effectively implementing these interventions, we have identified clusters of socially vulnerable neighborhoods whose residents are at a significantly higher risk of metastatic cancer at diagnosis. Interventions tailored toward these at‐risk neighborhoods that address social barriers of both insured and uninsured patients can potentially improve the rates of early‐stage diagnosis and overall cancer survival.

This study has several limitations. First, while we have identified significant associations, we cannot make causal inferences from this methodology. While we control for healthcare utilization, we are unable to control for the availability of other healthcare resources like diagnostic services. SVI measures average census tract vulnerability, which may not entirely reflect the patient‐specific vulnerabilities. In addition, SVI can change overtime as the population characteristics change. Future studies may show if the changes in neighborhood SVI mirror changes in cancer care for the patients residing there. Other social factors, in addition to the 16 that have been included in this study, may also be associated with the outcome. While the data sources used in this study maintain high standards, limitations of a database‐based and retrospective study apply. These include the discrepancies in the type of insurance patients have at the time of diagnosis which may result in measurement error and possible bias.

## 5 Conclusion

In addition to multiple demographic factors, increased social vulnerability was found to be associated with metastatic cancer at the time of diagnosis. Moreover, the association of SVI was seen to be present across various insurance types including those who were privately insured. By applying geospatial methods, we identified clusters where tailored interventions aimed toward at‐risk populations can potentially result in early diagnosis with improved survival.

## Author Contributions

**Muhammad Sohaib Khan:** conceptualization (lead), data curation (lead), formal analysis (lead), investigation (equal), software (lead), writing – original draft (lead). **Tammy Leonard:** conceptualization (supporting), formal analysis (equal), investigation (supporting), methodology (supporting), supervision (equal), writing – review and editing (lead). **Sean Young:** formal analysis (supporting), investigation (supporting), software (supporting), validation (supporting), visualization (equal), writing – review and editing (supporting). **Natalie Williams:** conceptualization (supporting), formal analysis (supporting), methodology (supporting), project administration (supporting), writing – review and editing (supporting). **Jennie Meier:** conceptualization (supporting), formal analysis (supporting), investigation (supporting), methodology (supporting), writing – review and editing (supporting). **Gilbert Z. Murimwa:** conceptualization (supporting), formal analysis (supporting), investigation (supporting), methodology (supporting), writing – review and editing (supporting). **Herbert J. Zeh 3rd:** conceptualization (supporting), data curation (supporting), investigation (supporting), methodology (supporting), writing – review and editing (supporting). **Patricio M. Polanco:** conceptualization (supporting), data curation (supporting), formal analysis (supporting), funding acquisition (lead), investigation (supporting), methodology (supporting), resources (lead), supervision (lead), visualization (equal), writing – review and editing (equal).

## Funding

The project was supported by the Eugene Frenkel Award, UT Southwestern Medical Center Harold C. Simmons Comprehensive Cancer Center. It was used for the salary of the research fellows involved in this project.

## Ethics Statement

The study was conducted after the approval of the University of Texas Southwestern Medical Center Institutional Review Board.

## Conflicts of Interest

Dr. Zeh reports that he is on the Scientific Advisory Board of Surgical Safety Technologies. Dr. Polanco reports that he is a Consultant at Iota Bioscience and Proctor at Intuitive Surgical. The rest of the authors have no related conflicts of interest to declare.

## Supporting information


**Appendix S1:** Supporting Information.

## Data Availability

The data underlying this article are available in the California Cancer Registry, at https://www.ccrcal.org/retrieve‐data/ and the Texas Cancer Registry, at https://www.dshs.texas.gov/texas‐cancer‐registry/data‐requests‐tcr.
