# Supplementary material for: Association of Neighborhood Social Vulnerability With Metastatic Cancer at Diagnosis
Source: Cancer Med. 2026 Jan 18;15(1):e71426. doi: 10.1002/cam4.71426 (PMC12813268; doi:10.1002/cam4.71426)
Supplement: Supplementary file 1 — Appendix S1: Supporting Information. [file CAM4-15-e71426-s001.docx]

**Supplementary Material for Review:**

Figure S1: Flow diagram of patients included and excluded.


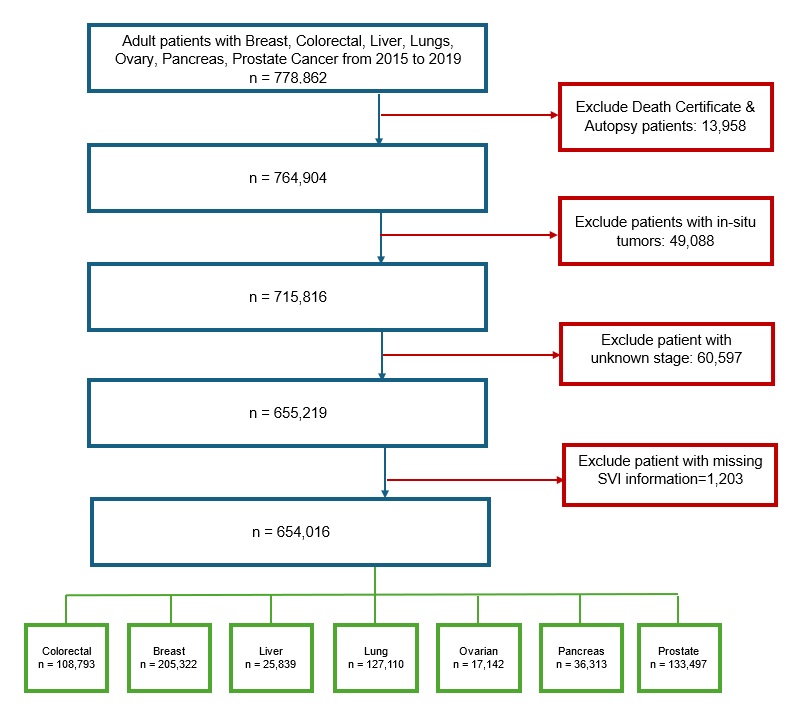


| Variables | Overall | Breast | Colorectal | Liver | Lung | Ovary | Pancreas | Prostate |
| --- | --- | --- | --- | --- | --- | --- | --- | --- |
| Person below 150% of Federal Poverty Level | 1.01 (1.004 – 1.01) | 1.02 (1.00 – 1.03) | 1.002 (0.99 – 1.01) | 1.03 (1.005 – 1.06) | 0.99 (0.99 – 1.006) | 1.01 (0.98 – 1.04) | 0.99 (0.98 – 1.01) | 1.002 (0.99 – 1.01) |
| Number of Persons unemployed | 1.001 (0.99 – 1.003) | 1.002 (0.99 – 1.009) | 1.003 (0.99 – 1.009) | 1.00 (0.99 – 1.01) | 0.99 (0.99 – 1.003) | 1.002 (0.99 – 1.01) | 1.003 (0.99 – 1.01) | 1.002 (0.99 – 1.01) |
| Number of units with housing cost burden | 1.004 (1.0003 – 1.007) | 0.99 (0.98 – 1.004) | 1.007 (0.99 – 1.01) | 1.00 (0.98 – 1.02) | 1.002 (0.99 – 1.01) | 0.99 (0.97 – 1.008) | 1.007 (0.99 – 1.02) | 1.006 (0.99 – 1.02) |
| Persons with no high school diploma | 1.03 (1.02 – 1.03) | 1.02 (1.01 – 1.03) | 1.003 (0.99 – 1.01) | 1.02 (0.99 – 1.04) | 1.006 (0.99 – 1.01) | 1.02 (0.99 – 1.04) | 1.007 (0.99 – 1.02) | 1.05 (1.03 – 1.07) |
| Persons uninsured | 1.006 (1.003 – 1.01) | 1.02 (1.01 – 1.03) | 1.003 (0.99 – 1.01) | 1.01 (0.99 – 1.03) | 1.004 (0.99 – 1.01) | 0.99 (0.98 – 1.02) | 1.009 (0.99 – 1.02) | 1.001 (0.99 – 1.01) |
| Persons aged 65 and older | 0.98 (0.98 – 0.99) | 0.99 (0.97 – 0.99) | 0.99 (0.98 – 0.99) | 0.99 (0.98 – 1.02) | 0.99 (0.99 – 1.00) | 1.005 (0.99 – 1.02) | 0.99 (0.98 – 1.008) | 0.99 (0.98 – 0.99) |
| Persons aged 17 and younger | 0.99 (0.99 – 0.99) | 0.99 (0.98 – 0.99) | 0.99 (0.99 – 1.001) | 1.01 (0.99 – 1.03) | 1.001 (0.99 – 1.01) | 1.02 (1.003 – 1.03) | 0.99 (0.98 – 1.003) | 0.99 (0.98 – 0.99) |
| Persons with a disability | 1.01 (1.01 – 1.02) | 1.01 (0.99 – 1.02) | 1.00 (1.00 – 1.01) | 1.01 (0.99 – 1.03) | 0.99 (0.99 – 1.004) | 0.99 (0.98 – 1.01) | 1.005 (0.99 – 1.01) | 1.02 (1.009 – 1.03) |
| Single Parent household | 1.002 (0.99 – 1.005) | 0.99 (0.99 – 1.01) | 1.00 (0.99 – 1.01) | 0.99 (0.98 – 1.01) | 1.002 (0.99 – 1.007) | 0.99 (0.98 – 1.007) | 1.009 (0.99 – 1.02) | 1.00 (0.99 – 1.01) |
| Persons who speak less than well English | 0.99 (0.98 – 0.99) | 1.002 (0.99 – 1.01) | 0.99 (0.98 – 0.99) | 0.99 (0.97 – 1.02) | 0.99 (0.99 – 1.006) | 1.0006 (0.98 – 1.02) | 0.99 (0.98 – 1.01) | 0.98 (0.97 – 0.99) |
| Persons of racial or ethnic minorities | 1.002 (0.99 – 1.006) | 1.001 (0.99 – 1.01) | 1.00 (0.99 – 1.01) | 0.99 (0.97 – 1.01) | 1.003 (0.99 – 1.01) | 0.99 (0.97 – 1.01) | 1.01 (0.99 – 1.02) | 1.01 (1.001 – 1.03) |
| Housing in structures with 10 or more units | 0.99 (0.98 – 0.99) | 1.00 (0.99 – 1.008) | 0.99 (0.98 – 0.99) | 0.99 (0.98 – 1.01) | 0.99 (0.99 – 1.00) | 1.007 (0.99 – 1.01) | 0.99 (0.99 – 1.006) | 1.005 (0.99 – 1.01) |
| Mobile homes | 1.005 (1.003 – 1.006) | 1.002 (0.99 – 1.007) | 0.99 (0.99 – 1.003) | 0.99 (0.99 – 1.01) | 1.002 (0.99 – 1.005) | 1.002 (0.99 – 1.01) | 1.005 (0.99 – 1.01) | 1.002 (0.99 – 1.008) |
| Household with more people than rooms | 1.005 (1.002 – 1.008) | 0.99 (0.99 – 1.007) | 1.00 (0.99 – 1.01) | 1.004 (0.99 – 1.02) | 1.005 (0.99 – 1.01) | 1.005 (0.99 – 1.02) | 1.00 (0.99 – 1.01) | 1.005 (0.99 – 1.01) |
| Households with no vehicles | 1.002 (0.99 – 1.005) | 1.002 (0.99 – 1.01) | 1.00 (0.99 – 1.007) | 0.98 (0.97 – 0.99) | 0.99 (0.99 – 1.003) | 0.99 (0.98 – 1.01) | 0.99 (0.98 – 1.01) | 1.005 (0.99 – 1.01) |
| Persons in group quarters | 1.003 (1.001 – 1.004) | 1.002 (1.00 – 1.008) | 1.003 (0.99 – 1.007) | 1.00 (0.99 – 1.01) | 1.004 (1.001 – 1.007) | 1.00 (0.99 – 1.01) | 0.99 (0.99 – 1.004) | 1.006 (1.00 – 1.01) |

Table S1: Multivariable logistic regression analysis for metastatic cancer at time of diagnosis of all cancers combined and stratified by cancer type.

* Models adjusted for age, race, gender, Hispanic ethnicity, insurance status, US State of Diagnosis, RUCC, Mean prevalence of routine checkup within the past year in residential county

**Table S2**: Proportion of Patients with Metastasis by Cancer Type and Social Vulnerability Tertile:

| Primary Site | Number of Patients | Patients with metastasis | Neighborhood Vulnerability  (Tertile Groups of SVI) | | |
| --- | --- | --- | --- | --- | --- |
|  |  |  | Least Vulnerable | Less Vulnerable | Most Vulnerable |
| Overall* | 654,016 | 149,476 (22.9) | 47,561 (19.9) | 54,226 (23.5) | 47,689 (25.9) |
| Breast* | 205,322 | 12,016 (5.8) | 3,894 (4.8) | 4,261 (6.0) | 3,861 (7.3) |
| Colorectal* | 108,793 | 25,415 (23.4) | 7,708 (21.9) | 9,147 (23.5) | 8,560 (24.6) |
| Liver* | 25,839 | 4,399 (17.0) | 921 (14.8) | 1,472 (16.7) | 2,006 (18.6) |
| Lung* | 127,110 | 65,831 (51.8) | 20,613 (49.7) | 24,633 (51.4) | 20,585 (54.6) |
| Ovarian | 17,142 | 9,269 (54.1) | 3,244 (53.3) | 3,241 (54.9) | 2,784 (54.0) |
| Pancreas* | 36,313 | 19,176 (52.8) | 6,718 (51.1) | 6,747 (52.8) | 5,711 (54.9) |
| Prostate* | 133,497 | 13,370 (10.0) | 4,463 (8.1) | 4,725 (10.3) | 4,182 (12.9) |

* Difference across tertile groups is statistically significant for all cancers with p-value <0.001 except for Ovarian cancers where the difference is not statistically significant.

Table S3: Adjusted* Odds of Metastatic Cancer at Diagnosis for every 10 unit increase in SVI for patients from California.

| Variables | All cancers combined | Breast | Colorectal | Liver | Lung | Ovary | Pancreas | Prostate |
| --- | --- | --- | --- | --- | --- | --- | --- | --- |
| SVI | 1.05 (1.05 – 1.05) | 1.04 (1.03 – 1.05) | 1.01 (1.01 – 1.02) | 1.04 (1.02 – 1.06) | 1.02 (1.01 – 1.03) | 1.01 (0.99 – 1.02) | 1.02 (1.01 – 1.03) | 1.07 (1.06 – 1.08) |

* Models adjusted for age, race, gender, Hispanic ethnicity, insurance status, RUCC, Mean prevalence of routine checkup within the past year in residential county

Table S4: Adjusted* Odds of Metastatic Cancer at Diagnosis for every 10 unit increase in SVI for patients from Texas.

| Variables | All cancers combined | Breast | Colorectal | Liver | Lung | Ovary | Pancreas | Prostate |
| --- | --- | --- | --- | --- | --- | --- | --- | --- |
| SVI | 1.04 (1.04 – 1.04) | 1.04 (1.03 – 1.05) | 1.01 (1.004 – 1.02) | 1.02 (1.01 – 1.04) | 1.01 (1.001 – 1.01) | 1.01 (0.99 – 1.03) | 1.01 (1.001 – 1.03) | 1.06 (1.04 – 1.07) |

* Models adjusted for age, race, gender, Hispanic ethnicity, insurance status, RUCC, Mean prevalence of routine checkup within the past year in residential county

Table S5: Multivariable logistic regression analysis for metastatic cancer at time of diagnosis of all cancers combined.

| Variables | | Overall | Overall  With Interaction of SVI and Insurance |
| --- | --- | --- | --- |
| SVI | | 1.04 (1.04 – 1.05) | 1.05 (1.05 – 1.06) |
| Age | Less than 50  50 to 64  65 to 79  80 or more | Ref  1.23 (1.19 – 1.26)  1.39 (1.37 – 1.44)  2.25 (2.19 – 2.32) | Ref  1.23 (1.19 – 1.26)  1.39 (1.36 – 1.43)  2.24 (2.18 – 2.30) |
| Gender | Male  Female  Non-Binary | Ref  0.76 (0.75 – 0.77)  0.69 (0.44 – 1.09) | Ref  0.76 (0.75 – 0.77)  0.69 (0.44 – 1.09) |
| State of Diagnosis | California  Texas | Ref  0.99 (0.98 – 1.01) | Ref  0.99 (0.98 – 1.01) |
| Race/Ethnicity | Non-Hispanic White  Non-Hispanic Black  Non-Hispanic Asian  Non-Hispanic/Other Races  Hispanic  Unknown Race/Ethnicity | Ref  1.06 (1.04 – 1.09)  1.06 (1.04 – 1.09)  0.98 (0.92 – 1.04)  0.83 (0.82 – 0.85)  0.18 (0.16 – 0.21) | Ref  1.06 (1.04 – 1.08)  1.06 (1.04 – 1.08)  0.97 (0.92 – 1.04)  0.84 (0.83 – 0.85)  0.18 (0.16 – 0.21) |
| Insurance Status | Private  Uninsured  Medicaid  Medicare  VA/Tricare/Military  Insurance NOS  Unknown/Missing | Ref  2.54 (2.46 – 2.62)  1.87 (1.83 – 1.92)  1.28 (1.26 – 1.29)  1.09 (1.04 – 1.15)  1.11 (1.07 – 1.15)  0.95 (0.91 – 0.98) | Ref  3.54 (3.29 – 3.81)  2.39 (2.26 – 2.52)  1.31 (1.27 – 1.35)  1.12 (1.01 – 1.24)  1.18 (1.10 – 1.27)  0.93 (0.86 – 1.00) |
| Insurance Status Interacted with SVI | Private  Uninsured  Medicaid  Medicare  VA/Tricare/Military  Insurance NOS  Unknown/Missing | - | Ref  0.94 (0.93 – 0.95)  0.96 (0.95 – 0.97)  0.99 (0.99 – 0.99)  0.99 (0.97 – 1.01)  0.98 (0.97 – 0.99)  1.00 (0.99 – 1.02) |
| Rural Urban Continuum Codes (RUCC) | RUCC 1  RUCC 2, 3  RUCC 6 - 9 | Ref  1.01 (1.00 – 1.03)  1.13 (1.11 – 1.16) | Ref  1.01 (0.99 – 1.02)  1.13 (1.10 – 1.16) |
| Mean prevalence of routine checkup within the past year in residential ZIP Code* | | 0.99 (0.98 – 0.99) | 0.99 (0.98 – 0.99) |

*PLACES 2020 Data at the ZIP Code Level

Table S6: Multivariable logistic regression analysis for metastatic cancer at time of diagnosis of all cancers combined.

| Variables | | Overall | Overall  With Interaction of SVI and Insurance |
| --- | --- | --- | --- |
| SVI | | 1.04 (1.04 – 1.04) | 1.05 (1.04 – 1.05) |
| Age | Less than 50  50 to 64  65 to 79  80 or more | Ref  1.23 (1.20 – 1.26)  1.40 (1.37 – 1.44)  2.26 (2.20 – 2.33) | Ref  1.23 (1.19 – 1.26)  1.39 (1.36 – 1.43)  2.24 (2.19 – 2.32) |
| Gender | Male  Female  Non-Binary | Ref  0.76 (0.75 – 0.77)  0.71 (0.46 – 1.12) | Ref  0.76 (0.75 – 0.77)  0.71 (0.46 – 1.11) |
| State of Diagnosis | California  Texas | Ref  1.00 (0.99 – 1.02) | Ref  1.00 (0.99 – 1.02) |
| Race/Ethnicity | Non-Hispanic White  Non-Hispanic Black  Non-Hispanic Asian  Non-Hispanic/Other Races  Hispanic  Unknown Race/Ethnicity | Ref  1.06 (1.04 – 1.08)  1.06 (1.04 – 1.08)  0.98 (0.92 – 1.04)  0.83 (0.82 – 0.85)  0.18 (0.16 – 0.21) | Ref  1.06 (1.04 – 1.08)  1.06 (1.03 – 1.08)  0.97 (0.92 – 1.04)  0.84 (0.83 – 0.85)  0.18 (0.16 – 0.21) |
| Insurance Status | Private  Uninsured  Medicaid  Medicare  VA/Tricare/Military  Insurance NOS  Unknown/Missing | Ref  2.54 (2.46 – 2.63)  1.87 (1.83 – 1.92)  1.28 (1.26 – 1.30)  1.11 (1.05 – 1.16)  1.11 (1.07 – 1.15)  0.95 (0.92 – 0.99) | Ref  3.53 (3.28 – 3.79)  2.36 (2.24 – 2.49)  1.31 (1.27 – 1.35)  1.12 (1.01 – 1.25)  1.19 (1.11 – 1.27)  0.93 (0.86 – 0.99) |
| Insurance Status Interacted with SVI | Private  Uninsured  Medicaid  Medicare  VA/Tricare/Military  Insurance NOS  Unknown/Missing | - | Ref  0.94 (0.93 – 0.95)  0.96 (0.95 – 0.97)  0.99 (0.99 – 0.99)  0.99 (0.97 – 1.01)  0.99 (0.97 – 0.99)  1.00 (0.99 – 1.02) |
| Rural Urban Continuum Codes (RUCC) | RUCC 1  RUCC 2, 3  RUCC 6 - 9 | Ref  1.02 (1.00 – 1.03)  1.14 (1.11 – 1.16) | Ref  1.02 (1.00 – 1.03)  1.13 (1.10 – 1.16) |
| Mean prevalence of routine checkup within the past year in residential census tract* | | 0.99 (0.98 – 0.99) | 0.99 (0.99 – 0.99) |

*PLACES 2024 Data at the Census Tract Level
